# A commentary on ‘Approaches of laparoscopic anatomical liver resection of segment 8 for hepatocellular carcinoma: a retrospective cohort study of short-term results at multiple centers in China’

**DOI:** 10.1097/JS9.0000000000000967

**Published:** 2023-12-18

**Authors:** Qingyun Xie, Fengwei Gao

**Affiliations:** a Department of Hepato-Pancreato-Biliary Surgery, People’s Hospital of Leshan, Leshan; b Liver Transplantation Center, State Key Laboratory of Biotherapy and Cancer Center, West China Hospital, Sichuan University and Collaborative Innovation Center of Biotherapy, Chengdu, People’s Republic of China

*Dear Editor,*

We have read with great interest the recent study by Wang *et al*.^1^ on the short-term efficacy of different surgical approaches for laparoscopic anatomical liver resection of hepatic segment 8 (LALR-S8) for hepatocellular carcinoma. The authors conducted a retrospective analysis of data on LALR-S8 from seven centers in China, including a total of 122 patients. They concluded that LALR-S8 is a safe and feasible treatment option for hepatocellular carcinoma, with the hepatic vein-guided approach being the most commonly used. While this study has important clinical implications, there are still several issues that require further discussion.

Firstly, the article does not mention whether paired analysis was considered to reduce potential confounding factors. When making multiple comparisons among groups, it is recommended to use analysis of variance or nonparametric tests for overall comparison rather than solely comparing two groups in order to avoid issues associated with multiple comparisons. If statistical differences exist after intergroup comparison, post hoc analyses such as the LSD (Least Significant Difference) test or Bonferroni correction should also be performed to determine which specific groups differ statistically^2,3^. It is important to provide detailed data of statistical tests, including degrees of freedom, test statistics, *P* values, etc., rather than using the vague expression of ‘statistically significant difference.’

Secondly, regarding the anatomy of hepatic veins in LALR-S8, Taniai *et al*.^4^ analyzed computed tomography data of 151 patients with tumors located in S8. They found that ~68.9% of cases had the typical anterior fissure vein (AFV), as shown in Fig. 1 of the article. Approximately 9.9% of cases had no AFV, and another 9.9% of cases had AFV originating from the root of the right hepatic vein and then running between the dorsal and ventral sections of S8. This indicates that AFV has a relatively high probability of variation. Accurately identifying the AFV is crucial for the complete resection or subsegmentectomy of S8 using the hepatic vein-guided approach^5^. Furthermore, it is important to address the issue of discerning the boundary between S5 and S8. According to the study by Li *et al*.^6^, ~75.4% of patients have intersegmental veins (ISV) between S5 and S8, although they were not identified in Fig. 1. Additionally, there was no discussion on how to avoid damaging the ISV between S5 and S8 via the hepatic vein approach to protect venous drainage of S5. Therefore, it is crucial to emphasize the development of individualized surgical plans based on preoperative imaging in order to preoperatively plan the anatomy of ISV and AFV and intraoperatively identify these important hepatic veins using ultrasound localization techniques.

Finally, regarding the surgical approach, it should be noted that the claim made in this article about the frequency of signature hepatic veins on the fluorescent-guided intrahepatic plane being 100%, which indicates that there is no contradiction between them may be influenced by selection bias in patient recruitment, as this study was retrospective. Therefore, it is important to avoid making overly absolute claims. While numerous anatomical studies^7^ support the classic Couinaud segmentation scheme, which suggests that the right boundary of S8 corresponds to the middle hepatic vein (MHV), corrosion cast studies have shown that the dorsal interface of S8 does not necessarily align with the right hepatic vein (RHV) in many cases. Hence, it is crucial to prioritize adhering to the fluorescent interfacial boundary for parenchymal transection as a surgical principle rather than solely focusing on exposing the RHV.

The authors’ comprehensive research and analysis on LALR-S8 is highly appreciated. However, there is a need for further refinement in future studies by adopting a more rigorous research methodology, study design, and statistical analysis. This would enhance the credibility and explanatory power of similar studies.

## Ethical approval

Not applicable.

## Consent

Not applicable.

## Source of funding

Not applicable.

## Author contribution

Q.X.: study design and writing; F.G: critical review and study supervision.

## Conflicts of interest disclosure

The authors declare no conflict of interest.

## Research registration unique identifying number (UIN)

1. Name of the registry: not applicable.
2. Unique identifying number or registration ID: not applicable.
3. Hyperlink to your specific registration (must be publicly accessible and will be checked): not applicable.

## Guarantor

Fengwei Gao

## Date availability statement

All data generated or analyzed during this study are included in this article. The data are available from the corresponding author upon reasonable request.

## Provenance and peer review

This paper is not commissioned and externally peer-reviewed.
